# Climate change, future warming, and adaptation in Europe

**DOI:** 10.1093/af/vfy036

**Published:** 2019-01-03

**Authors:** Massimiliano Pasqui, Edmondo Di Giuseppe

**Affiliations:** 1 Institute of Biometeorology – National Research Council (CNR – IBIMET), Rome; 2 Dipartimento di Scienze Bio-Agroalimentari – National Research Council (CNR – DISBA), Rome

**Keywords:** heat waves, impacts, perception, vulnerability

**Implications**

- In recent decades, the increased temperatures reported in Europe and in the Mediterranean basin represent one of the clearest footprints of climate change along with increased frequency of heat waves.
- These climate modifications put the environment and human activities under strong pressure with a resulting need for designing new adaptation and mitigation strategies.
- The climate change challenge is unprecedented for humanity and is recognized as a priority topic for future research. Changes in the way we think and behave are critical challenges at the global and regional levels.

## Introduction

Climate change is a fundamental challenge for humanity as it deeply and pervasively affects the way we live on the planet. All human activities are affected by climate variability, which is due to natural factors (changes of natural cycles of atmospheric and oceanic mechanisms) and anthropic activities (greenhouse gas production). Climate change has an extremely heterogeneous character in terms of space, temporal variability, and distribution. This peculiarity implies the need to identify key local factors for the geographical area of interest along with knowledge of remote forces and an effective multidisciplinary approach to tackle its negatives impacts.

Climate change has been a relevant issue at the international level since the late 1980s with the creation of the Intergovernmental Panel on Climate Change by the United Nation General Assembly (Resolution 43/53, 1988). Subsequently, the Intergovernmental Panel on Climate Change First Assessment Report (IPCC, 1990) stated, “ … *there is a natural greenhouse effect which already keeps the Earth warmer than it would otherwise be; emissions resulting from human activities are substantially increasing the atmospheric concentrations of the greenhouse gases carbon dioxide, methane, chlorofluorocarbons and nitrous oxide. These increases will enhance the greenhouse effect, resulting on average in an additional warming of the Earth’s surface*” (IPCC, 1990). This large scale and organized scientific assessment provided the initial basis for the interpretation a deep modification of the earth’s climate system. During the past three decades, more assessments of climate change have been produced by the Intergovernmental Panel on Climate Change, all of them drawn on the work of hundreds of scientists from around the world (IPCC, 2013, 2014a, 2014b).

The phase of intense global warming we experienced in recent decades began unequivocally in the 1950s and has accelerated since the 1980s (IPCC, 2014a, 2014b; Baldi et al., 2006; Zampieri et al., 2016). This increase affected both the average monthly temperature and seasonal values along with extreme climate events (IPCC, 2014a, 2014b).

## Global Warming and Heat Waves

**Figure 1.**
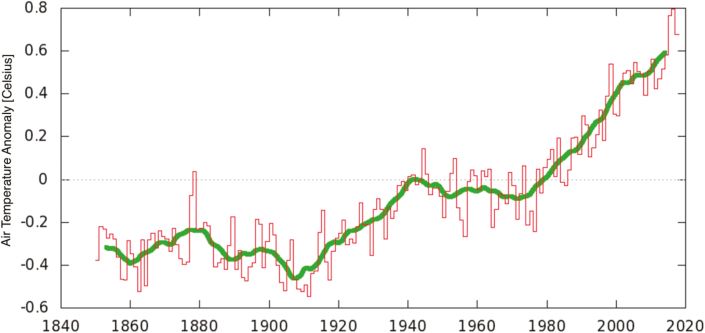
Annual global mean air temperature anomaly (°C) at the surface (Jan–Dec), based on the HadCRUT4 global temperature dataset (https://crudata.uea.ac.uk/cru/data/temperature/). The time series is computed with the KNMI Climate Explorer.

Global warming (Figure 1) produces effects that are measurable through physical indicators such as rising sea levels, increased heat content of the oceans, decreased snow and ice surface coverage (both marine and terrestrial), and increased frequency of very hot days and of very intense rains (IPCC, 2014b). Among these climate change features, extreme events are largely relevant for assessing impacts and defining coping options. For simplicity, an extreme event is defined as a climate event in which the related physical values overpass a threshold which is closed to the extreme possible values for that variable (IPCC, 2012).

In this regard, a collection of 27 weather-climatic indicators were established to identify the occurrence of extreme events for monitoring purposes and for future projections of climate (Sillmann et al., 2013a, 2013b).

**Figure 2.**
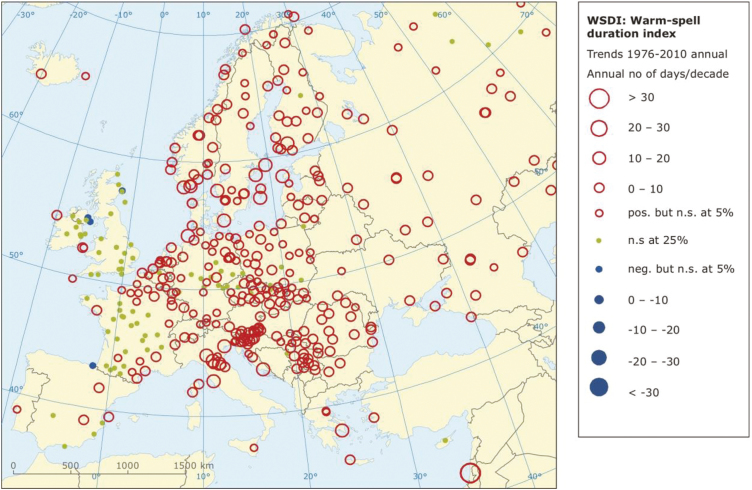
Warm spell duration index computed trends for 1976–2010. Circles represent the annual mean number of days for the decade. Map is from European Climate Assessment and Dataset E-OBS gridded dataset (https://www.ecad.eu).

**Figure 3.**
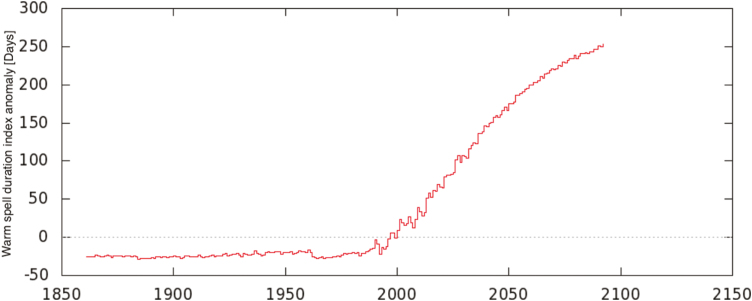
Warm spell duration index annual anomaly for the historical + RCP8.5 ensemble mean CMIP5 future climate scenarios (with respect to the 1981–2010 reference period). The WSDI index is averaged over the Europe geographical domain (−10°E–45°E and 35°N–65°N). Only land grid points have been taken into account. In the vertical axis, the annual mean anomalous number of WSDI-ETCCDI days is shown. In the horizontal axis, time span is shown. Data and computation from the KNMI Climate Explorer.

Projections for the 21st century by the 27 member Expert Team on Climate Change Detection and Indices indicators carried out on the basis of different climate models and different carbon dioxide emission scenarios indicate an increase in the frequency of extremely hot days and an increased number of consecutive hot days (Sillmann et al., 2013b) as shown in Figures 2 and 3.

There are some characteristics of the climate change footprint that exhibit a more extensive nature. In Europe, among these climate change footprints, we must certainly highlight the increase in summer temperatures and a coherent increase of hot days and heat waves (Zampieri et al., 2016). There is no specific definition of a heat wave; each heat wave arises from the need to characterize the effects of the increase in temperature for long periods in a special specific sector of interest such as human health, crop production, livestock production, and the environment. Certainly, heat waves are relevant for all aspects that are intrinsically linked to factors of “suffering from heat,” to which living beings in general are subjected (McMichael et al., 2006; Lacetera et al., 2013; Özkan et al., 2016). These extreme periods are referenced as high-impact weather events, along with other completely different events such as floods, wind storms, or cold waves. Heat waves could be classified according to their duration and/or their intensity which is measured by the amount that the recorded air temperature deviates from the reference climatological values. It is the combination of these features, duration and intensity, which determines effects on human activities and on the health of animals.

Based on the indications provided by the Expert Team on Climate Change Detection and Indices working group, a heat wave can be defined as the phenomenon for which there is a sequence of at least 6 days with maximum daily temperature or temperature daily minimum above the corresponding daily threshold value at the 90th percentile (Karl et al., 1999). More specifically, it is calculated as a series of daily values for the analysis period, in such a way as to have a specific threshold value for each observation day and thereafter the threshold is exceeded day by day. Thus, air temperature values during the heat wave are considerably higher than the reference climate values ​​for that period and for that geographical area.

To characterize heat waves, the Expert Team on Climate Change Detection and Indices group defined the Warm Spell Duration Indicator index as the number of days that belong in a heat wave. Starting from weather station data, using model reanalysis and modeling future climate scenarios, it is possible to reconstruct the trends of daily air temperature anomalies and to identify hot days and heat waves.

What do we know about heat waves? In Europe, scientific studies and sector reports indicate a clear trend of an increasing number of hot days together with an increase in warm periods and heat waves (Baldi et al., 2006; Zampieri et al., 2016). The Mediterranean area is, therefore, a climate change hot spot (Giorgi, 2006), since it stands out for being one of the most critical areas for heat waves and related issues (Baldi et al., 2006; Giorgi and Lionello, 2008, Efthymiadis et al., 2011; Ulbrich et al., 2012). This specific climate signal became more evident in the second half of the 20th century. At the same time, a number of studies on future climate projections indicate how the footprint of this extreme warming feature will persist in the future. It is indeed very probable that the frequency of hot days and heat waves will increase significantly in the future. Thus, intensification is likely (Figures 1–3).

It should also be emphasized that the opposite weather conditions, characterized by the number of cold days and the number of cold waves, exhibited a significant decrease in the last 30 yr and the same trend is expected to persist in the future in Europe and in the Mediterranean basin.

## Climate Change Impacts and Adaptation

In the last decades, an effort has been made by the scientific community to enhance our scientific knowledge of the fundamental mechanisms of the Earth’s climate system as well as the implications and impacts of climate change. A portion of this effort has been directed to identify the new actions for mitigating anthropogenic greenhouse gas emission trends. Other efforts have focused on identifying new actions to adapt to the observed and expected changes in climate (IPCC, 2013, 2014a, 2014b).

Thus, defining and designing salient actions to tackle the negative effects of climate change must be planned at the local scale to guarantee their effectiveness. To be legitimate, these actions must be developed in accordance with surrounding landscape structures and socioeconomic and environmental regional characteristics and, finally, in accordance with national and international policies.

Climate change modifies the specific thermo-physical features and frequency of occurrence of climatic events. Therefore, modification of air temperatures, precipitation amounts, air humidity levels, ventilation intensities, and occurrence of extreme events such as floods, drought, cold waves, and heat waves due to climate change produces impacts on the environment and on agricultural and livestock production systems. For these reasons, agriculture is one of the most vulnerable production sectors to the forces of climate variability and climate change.

Direct impacts of climate change on livestock can be identified. These include changes in eating behavior and changes in animal physiology. Indirect impacts of climate change on livestock are also apparent and include pathogen ecology, water resource quality, and increased mortality of individuals. Climate change also alters livestock agronomic practices and management strategies. The direct and indirect impacts of climate change are modulated by different factors such as geographical location, specific animal characteristics, the intensity of extreme events, and the level of exposure. Specific effects on animals include altered well-being, health, and conformation, which in turn have a direct effect on the quality and quantity of livestock production (Özkan et al., 2016).

Changes in the quality of livestock production force modifications on food safety, food availability, greenhouse gas emissions, and farm income variability may also have social impacts. In fact, this is the schematized and simplified process that leads to a potential change in the livestock sector from pure climatic variation. This complex network of interagent factors can be seen as an arena in which there is strong competition and potential conflict between the key factors (Köchy et al., 2017).

Climate change can modify the conditions in which farmers typically operate by introducing new levels of uncertainty, many of which were previously unknown. These complex and demanding conditions call for new motivations to adapt strategically and cope with climate change. These efforts are relevant to the complex field of livestock production, in particular, in southern European areas and in the Mediterranean (Segnalini et al., 2011), where the impact of climate change seems to be more evident and substantially negative (Dono et al., 2016). The increase in summer temperatures and the increase in number and intensity of heat waves together with a persistent reduction in water resources negatively affect dairy production. Indeed, recent studies have shown that heat waves lead to increased mortality rates in dairy cattle (Vitali et al., 2009) and a decrease in the quality and quantity of milk produced (Bertocchi et al., 2014). Therefore, climate change will have a significant economic impact on the income of the agricultural enterprise (Dono et al., 2013).

Management of livestock during heat waves is critical for livestock producers and will have an impact on the income of livestock producers. The negative effects of heat stress on livestock can be summarized as follows: 1) an increase in animal mortality rates, especially due to impaired immune responses and the spread of infectious diseases, 2) reduced fertility due to altered hormonal patterns, 3) reduced feed intake and growth rates, and 4) reduced amounts of milk, especially in high-producing dairy cows.

Furthermore, climate and environmental changes associated with high temperatures, high levels of carbon dioxide, and modification of rainfall frequency will likely affect crop production, which is fundamental for the feed and forage supply for livestock. The direct effects of climate warming and reduced rainfall are reductions in feed and forage yields, alteration of nutritional value (e.g., increased lignification), and variation of the floristic composition of the biomass. Indirect effects of climate change include diffusion of parasites and pathogens as well as increased invasiveness of some plant species. The loss of biodiversity and deteriorated soil functions due to extreme climate events must be considered within the big picture of the challenges of climate change.

## Perceptions of Climate Change

In recent decades, robust scientific knowledge has been produced that provides important information that can be used to make science-based decisions. However, additional decision-support tools and an understanding of the cognitive processes associated with perceptions of climate change are needed to use this information to transform society to be resilient to climate change.

**Figure 4.**
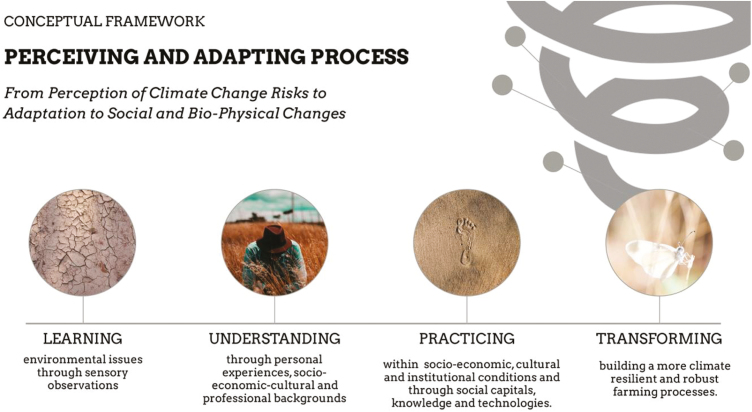
The cognitive process: a conceptual framework of perceiving and adapting process (adapted from Nguyen et al., 2016a).

The conceptual reference framework of this cognitive process of the perception to climate adaptation (Figure 4) can be divided into several, related phases (Nguyen et al., 2016a, 2016b) as follows:

1. The first phase is when the farmer learns about local, environmental aspects through direct observations.
2. The second phase is completed when the farmer understands, through direct experience, the economic, professional, social, and cultural backgrounds of the area in which he/she operates.
3. The third phase consists of practice in a specific socioeconomic, social, cultural, and institutional setting of conditions. This stage is also enriched by social, scientific, and technological knowledge that the farmer could borrow from personal and institutional relationships.
4. The final phase is reached when there is effective transformation of decision-making processes toward a state of greater resilience and robustness with respect to climate change.

The first two phases are driven by the farmer’s personal adaptation, which is modulated by the perception of information related to risks associated with climate change. The last two phases are represented by the farmers’ ability to adapt and change, which comes from biophysiological and social processes. For these reasons, the process of adaptation to climate change must be built on both dimensions of learning for adapting (“perceiving to learn and to adapt” and “learning to perceive and to adapt”) in order to sustain adaptive response cycles to climate change (Nguyen et al., 2016b).

## Conclusions

The challenge of climate change is unprecedented for humanity and requires a significant change in our way of thinking and acting (IPCC, 2014b). We now know, with a heterogeneous, but reasonable level of reliability, how future climate change scenarios will affect agro-ecosystems, landscapes, coastlines, agricultural yields, and local and global economies. However, how these changes will affect society, in general, are still not known.

To develop an effective climate change adaptation strategy, scientists, citizens, farmers, livestock producers, and policy makers will need to adapt a new process of thinking and learning, which must be based on current scientific information. Adaptation to climate change must be implemented as a continuous transformation, which implies continuous change at different levels of society. Institutions also play a substantial role within this transformation process. Stakeholders must be aware of the potential negative impacts and threats associated with climate change and they must be willing to engage in debate to enhance their learning and to integrate scientific and traditional knowledge to develop and implement innovative adaptation strategies. In addition, there is need for additional public–private partnerships to deal with complex issues such as those related to human health and water governance to support nonlitigious mediation of environmental conflicts.

## About the Authors

**Massimiliano Pasqui** is a climate scientist. He graduated in physics and obtained a PhD in Physical Modeling for Environmental Protection. His main interests are the numerical modeling in support of weather forecasts, climate data analysis, seasonal forecast predictability, and climate change impacts. In the last decade, he has been engaged as a contract professor in meteorology, numerical modeling, and climatology courses at several Italian universities, as well as in post-lauream master classes organized by national and international research organizations.

**Edmondo Di Giuseppe** is a climate data analyst at CNR-DISBA (Dipartimento di Scienze Bio-Agroalimentari). His work bridges statistical methodology and software tools to analyze climate phenomena. His fields of interest in the framework of Statistical Climatology are clustering, extreme values, geostatistics, space-time models, and Bayesian inference.
